# Influence of time of day on resting motor threshold in clinical TMS practice

**DOI:** 10.1016/j.clinph.2023.08.017

**Published:** 2023-09-09

**Authors:** Karen Wendt, Majid Memarian Sorkhabi, Jacinta O’Shea, Timothy Denison, Joram van Rheede

**Affiliations:** a MRC Brain Network Dynamics Unit, Nuffield Department of Clinical Neurosciences, University of Oxford, Oxford, OX1 3TH, UK; b Department of Engineering Science, University of Oxford, Oxford, OX1 3PJ, UK; c Magstim Company Limited, Spring Gardens, Whitland SA34 0HR, UK; d Wellcome Centre for Integrative Neuroimaging, Oxford Centre for Human Brain Activity (OHBA), University of Oxford Department of Psychiatry, Warneford Hospital, Warneford Lane, Oxford, UK

Transcranial magnetic stimulation (TMS) is increasingly used in the clinic for the treatment of depression, OCD and other psychiatric conditions. However, there is large variability in clinical outcomes. Identifying the sources of such variability is important to ensure consistency of treatment response. Recent papers from the ‘Big TMS Data Collaboration’ examined potential sources of variability in neural responses to TMS (Corp et al., 2020), and identified time of day (TOD) as one important factor. Indeed, cortical excitability has been shown to be influenced by various factors that vary systematically with TOD, such as time awake and circadian phase (Ly et al., 2016).Yet, studies looking at measures of the neural response to TMS in healthy participants, including motor threshold and evoked responses, found no effect of TOD (e.g. (ter Braack et al., 2019)).

To calibrate TMS pulse intensity before applying therapy, the operator measures a patient’s individual resting motor threshold (RMT). This is defined as the minimal intensity at which a TMS pulse elicits a motor response. If RMT is measured at one clock time while clinical TMS treatment is delivered at another, TOD differences in cortical excitability could lead to discrepancies between the intended and achieved level of brain excitation and therefore treatment ‘dose’. As clinical outcomes in TMS may be dose-dependent (Tendler et al., 2023), this could have important implications.

Here, we take advantage of a large data set of RMTs from 60 TMS clinics across the United States to investigate the relationship between TOD and RMT in clinical practice. All data were gathered using the Horizon 3.0 system (Magstim Ltd, Wales) in accordance with HIPAA guidelines and Magstim’s agreements with each clinic. All stimulators had the same specifications with a biphasic pulse waveform and a figure-of-8 stimulation coil. The data set includes 2626 data points from 1550 patients, collected between October 2021 and March 2023. All patients were undergoing TMS treatment for major depressive disorder.

For each patient, only the most recent RMT was used in the analysis. Device time in UTC was converted to local clinic TOD using a US zip code database (https://simplemaps.com/data/us-zips). As patient metadata was only included in few records (gender: 191, ethnicity; 32, handedness: 28), only RMTs, local time and clinic were analysed here. Data points with RMTs more than 2.5 standard deviations away from the sample mean were removed, resulting in 1514 RMT measurements with a median of 57.0 % of the maximum stimulator output (MSO) of the Horizon 3.0 system (Figure 1a).

To evaluate the influence of TOD on RMTs, we fit the data according to TOD, and calculated the variance explained by this fit (Var_TOD_), similar to (van Rheede et al., 2022). The fit was computed using a moving average of the data with a window size of 60 minutes, with window centres ranging from 8:30-17:30 in 6-minute steps. We calculated the percentage of variance explained by time of day as follows:

(1) $${\text{\%Var}}_{\text{TOD}\;}\text{=}\left({\text{Var}}_{\text{total}\;}-{\text{Var}}_{\text{remaining}\;}\right){\text{/Var}}_{\text{total}\;}\text{*100}$$

Where Var_total_ is the variance of RMT in the data set and Var_remaining_ is the variance of the data set after subtracting the TOD fit estimate. Values for %Var_TOD_ were compared to a reference distribution generated through 10,000 shuffled versions of the data set (where time stamps were reassigned to RMT values randomly) to estimate the likelihood of obtaining such a value by chance.

TOD explained a significant percentage of the variance in the RMTs (Fig. 1b; 4.08%, p < 0.001 vs. shuffled). While prior work on the effect of time awake and circadian rhythm (Ly et al., 2016) on cortical excitability would predict a gradual *decrease* in RMT, RMT appears to increase throughout the working day, and shows fluctuations that could reflect, e.g., cortisol spikes.

However, apparent TOD effects could also arise through differences in average RMT and scheduling between clinics.We therefore compared scheduling and mean RMTs between the 10 largest clinics with at least 45 patient data points. Indeed, clinics had different distributions of measurement times across the working day (Fig. 1c) and a one-way ANOVA with factor Clinic showed that RMTs differed significantly between the 10 largest clinics (F(9, 729) = 83.09; p < 0.001; Fig. 1d).

To control for the effect of centre on Var_TOD_, RMTs from the 10 largest clinics were z-scored by clinic and outliers (|z-score| > 2.5) were removed. While %Var_TOD_ in this smaller sample (N = 739) before z-scoring was high and significant (11.07%, p < 0.001 vs. shuffled), %Var_TOD_ after controlling for clinics was greatly reduced and not significant (1.19%, p = 0.498, vs. shuffled; Fig. 1e). This indicates that the apparent effect of TOD was due to the differences between centres and their testing schedules.

Therefore, while prior work suggested that cortical excitability might show a predictable course over the working day, we did not find a significant effect of TOD on RMTs measured in the clinic. This corresponds with previous studies in healthy volunteers (e.g. (ter Braack et al., 2019)), which found no daytime changes in RMT. Notably, our initial analysis, without accounting for inter-centre variability, did suggest an effect of TOD; this highlights the importance of controlling for possible methodological differences between clinics when pooling TMS data from multiple centres.

It is possible that the large inter-individual variability in RMTs masked more subtle TOD effects in our analysis. Additionally, patients with depression could have altered cortical excitability time courses due to associated disruption of sleep and circadian factors or the effects of antidepressant medication. To fully rule out effects of TOD in clinical TMS, future studies should collect multiple RMT values per patient at time points spanning the working day to allow a within-subject analysis. Factoring in other variables, including age, gender, menstrual cycle and hormonal levels, will also be important to identify potential interactions with any effects of time of day on RMT. Nevertheless, the lack of a significant effect in this large data set suggests that any influence of TOD will be subtle.

## Figures and Tables

**Fig. 1:**
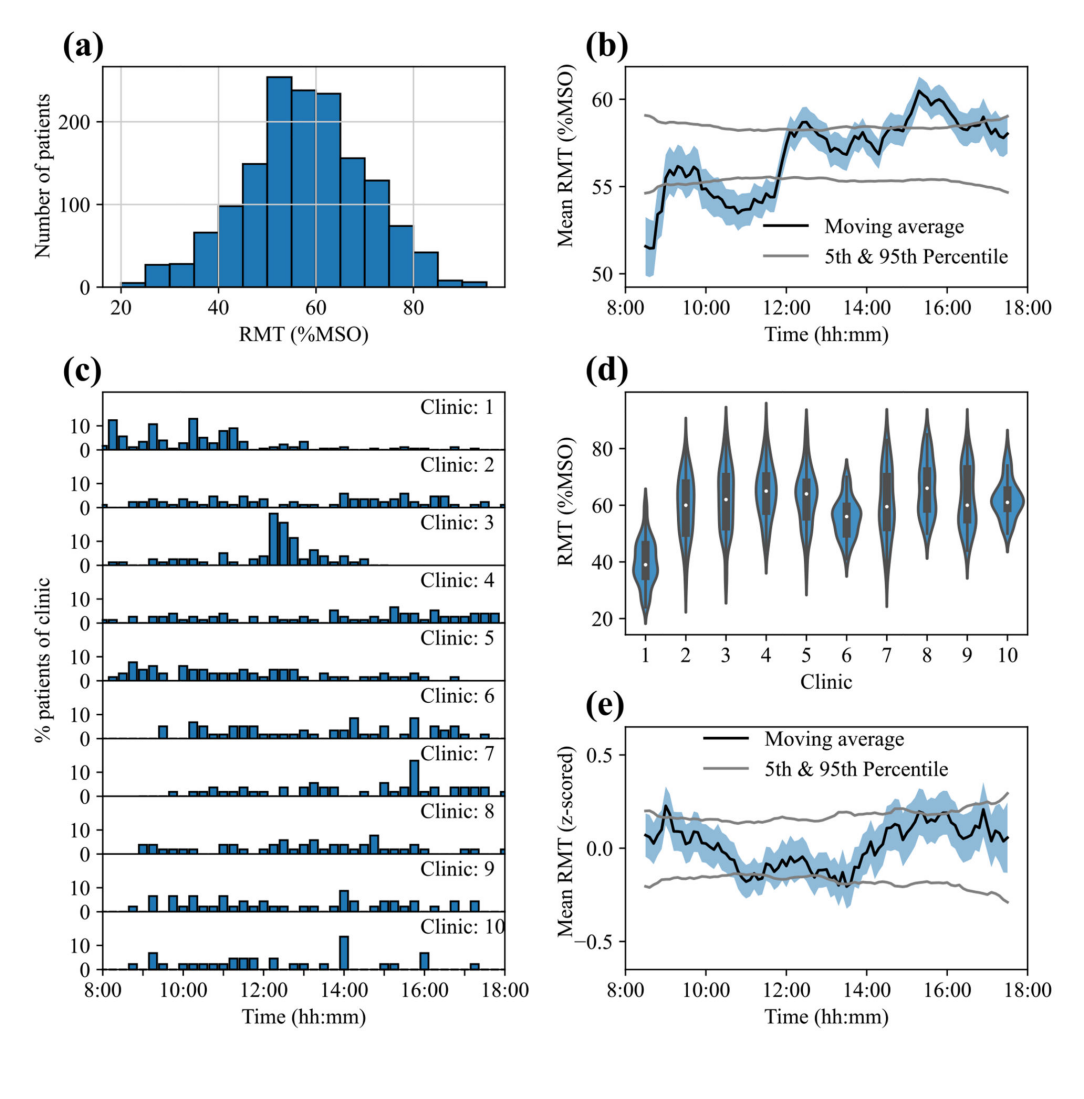
**(a)** Distribution of resting motor thresholds (RMTs) of all patients. **(b)** The moving average time of day fit of the RMT values is shown in black with its standard error represented by the shaded region in blue. The grey lines indicate the 5^th^ and 95^th^ percentile of the moving average calculated after shuffling the data 10 000 times, which represents the boundaries a moving average is likely to reach when no diurnal effects are present. **(c)** The measurement distributions across the day from 8 am – 6pm are shown for each clinic with at least 45 patients. Of these 10 clinics, some tend to schedule their patients at particular times of day as indicated by the distributions that are skewed to a specific time of day, e.g. clinic 1. **(d)** The distribution of RMT levels is shown for each of the 10 largest centres included in (c). **(e)** The moving average fit of the RMT values after controlling for site. The mean is shown in black with its standard error in blue and the 5^th^ and 95^th^ percentile obtained through temporal shuffling shown in grey.

## Data Availability

The data and relevant scripts will be made available upon request. Please contact Magstim Company for access to the data, and the corresponding author (KW) for access to the analysis code.
